# Factors Influencing Solid Waste Management Practices and Challenges in Awi Administrative Zone, Northwestern Ethiopia

**DOI:** 10.1155/bmri/1311674

**Published:** 2025-01-29

**Authors:** Belsti Atnkut, Atalaye Nigussie, Bulti Kumera, Alemu Tsega, Fekadu Tiruneh, Wondwosson Kibrie Minale, Fentanesh Anemut, Ewunetu Tazebew, Baye Terefe, Amare Fassil, Getaneh Gebeyehu, Tess Astatkie

**Affiliations:** ^1^Biology Department, College of Natural and Computational Science, Injibara University, Injibara, Ethiopia; ^2^Statistics Department, College of Natural and Computational Science, Injibara University, Injibara, Ethiopia; ^3^Department of NaRM, College of Agriculture, Food and Climate Science, Injibara University, Injibara, Ethiopia; ^4^Department of Forestry and Climate Science, College of Agriculture, Food and Climate Science, Injibara University, Injibara, Ethiopia; ^5^Department of Geography and Environmental Studies, College of Social Science and Humanities, Injibara University, Injibara, Ethiopia; ^6^Doctoral School of Biological Science, Hungarian University of Agriculture and Life Science, Gödöllő, Hungary; ^7^Faculty of Agriculture, Dalhousie University, Truro, Nova Scotia, Canada

**Keywords:** Awi zone, generation rate, public health, solid waste collection, solid waste management

## Abstract

**Background:** Effective management of solid waste, generated as a result of human activities, is crucial. However, improper solid waste management seriously threatens the environment and public health in developing countries including Ethiopia.

**Objective:** This study is aimed at assessing the status of solid waste management practices and identifying key factors in Awi Administrative Zone, Ethiopia.

**Methods:** A community-based cross-sectional study design was used to collect the data and then analyze using descriptive statistics and logistic regression modeling. The survey was conducted in select kebeles (wards) (administrative subdivisions) of Injibara, Dangila, and Chagni towns, using two-stage sampling techniques. Primary and secondary data sources were utilized. The per capita waste generation rate was calculated based on the total solid waste generated in kilograms per total family size of households per year.

**Results:** The per capita per day solid waste generation rates for Injibara, Dangila, and Chagni were 0.443, 0.456, and 0.487 kg/capita/day, respectively. The composition of household solid waste mainly consisted of biodegradable and nonbiodegradable materials. It was concluded that a significant proportion of household solid waste comprised biodegradable organic matter, which could be efficiently recycled or decomposed through microbial activity. Of the households, 40.6% had access to door-to-door solid waste collection service, and 35.9% and 26.2% of the households disposed their solid waste on riverside bridge/drainage lines and roadside/open land, respectively. The regression analysis showed that the head of the household's age, family size, monthly income, solid waste selling practice, solid waste reduction practice, awareness of solid waste disposal rules, frequency of household cleanup campaign participation, and awareness of the impacts of improper solid waste management on the environment and human health were significantly associated with improper solid waste management practices.

**Conclusion:** The study revealed poor performance in solid waste management in the study area, attributed to factors such as inadequate collection system design and schedule, open burning of refuse, substandard condition of the final dumpsite, and lack of community awareness leading to illegal dumping.

**Recommendations:** Based on the findings of the study, we recommend that the town municipality strengthen its door-to-door solid waste collection service and distribution of communal bin containers, conduct environmental assessments for better dumpsite selection, and implement a community-based waste management system to ensure sustainable solutions and continuous education on proper solid waste management practices.

## 1. Introduction

Efficiently handling waste has emerged as a significant issue in urban regions of developing nations. This presents a widespread environmental and public health concern on a global scale, jeopardizing the well-being of numerous urban communities [1]. Globally, the quantity of solid waste is increasing more quickly than urbanization. In 2012, cities produced 1.3 billion tons of solid waste annually, with predictions indicating an increase to 2.2 billion tons by 2025 [2]. Contributing factors include rapid population growth, unintended development, changes in consumption patterns, and inadequate recycling and reuse practices [3, 4]. Currently, urban areas generate 2.3 billion tons of solid waste yearly, with forecasts suggesting a rise to 3.4 billion tons by 2050 [5]. Sub-Saharan Africa generates around 62 million tons of solid waste annually, yet waste collection rates in developing nations remain below 70% [6].

The main potential risks associated with improper solid waste management (SWM) practices or poorly managed disposal sites are soil and water contamination, fire incidents, malarial disease occurrences, and other related issues [3, 7]. In developing nations like Ethiopia, improper SWM is widespread in many cities where solid waste is dumped in unapproved locations such as roadside ditches, drainage systems, and open areas which causes environmental pollution and triggers disease outbreaks in city areas [3, 8, 9].

Studies show that only 30%–50% of the waste generated in developing countries is collected and managed properly [10]. The remaining portion is either incinerated or permitted to undergo decomposition in unenclosed areas or is disposed of in unregulated landfills, actions that pose a threat to the natural surroundings. In the continent of Africa, a small portion of the solid waste produced is retrieved, and an overwhelming majority of that quantity, amounting to 95%, is commonly discarded either in the vicinity of urban perimeters or in provisional locations, usually vacant spaces scattered across the urban landscape [11]. While cities are producing an ever-growing volume of waste, the effectiveness of their solid waste collection and disposal systems is declining. The insufficient management of solid waste is the cause of pollution affecting the environment and the health of living things in the cities, and it is one of the most critical environmental problems that cities and towns are facing in the continent. The current capacity of most SWM systems in Africa is inadequate and too slow to meet the increasing demand for proper SWM [12].

Ethiopia's SWM policies, guided by the Environmental Policy and the Solid Waste Management Proclamation, emphasize integrated waste management approaches, community participation, and recycling, while facing challenges such as inadequate infrastructure, limited funding, and varying practices between urban and rural areas [13]. SWM poses a significant predicament for developing nations, such as Ethiopia, due to the potential social, economic, and environmental ramifications that can arise if not adequately addressed [8, 13]. Poorly managed solid waste harms health, the local and global environment, and the economy of Ethiopia [14]. Its negative influence on health includes the spread of communicable diseases, encompassing bacterial, viral, and other ailments. Poorly managed solid waste also serves as a breeding ground for insects such as mosquitoes, which are responsible for transmitting protozoan diseases. In tropical climates, certain airborne insects are directly associated with the spread of indigenous diseases. Moreover, the accumulation of uncollected waste obstructs drainage channels and exacerbates health issues linked with stagnant water in ponds. Furthermore, accumulated waste poses a risk to individuals, particularly children, who come close to it, as there is a constant threat of physical harm [15].

The composition, quantity, and amount of waste generated in Awi administrative zone is alarmingly increasing from time to time mainly due to the fast growth of population and rapid urbanization [16]. The Awi Administrative Zone faces insufficient waste collection and disposal infrastructure, resulting in increased littering and illegal dumping, compounded by low collection rates, limited public awareness of proper waste management practices, and resource constraints that hinder local governments from implementing effective systems. Although SWM is considered one of the most pressing public health issues in the Awi Administrative Zone, there has been no study conducted to assess the scale of the problem or gather limited information about the factors associated with and challenges of household-based SWM practices in the area. Therefore, this study is aimed at assessing the status of SWM practices and identifying its associated factors in Awi Administrative Zone, Ethiopia. The findings of this study may assist urban planners, health workers, and researchers interested in waste management in focusing on solid waste collection, sorting, and disposal practices as the foundation for sustainable waste management.

## 2. Materials and Methods

### 2.1. Study Area

**Figure 1:**
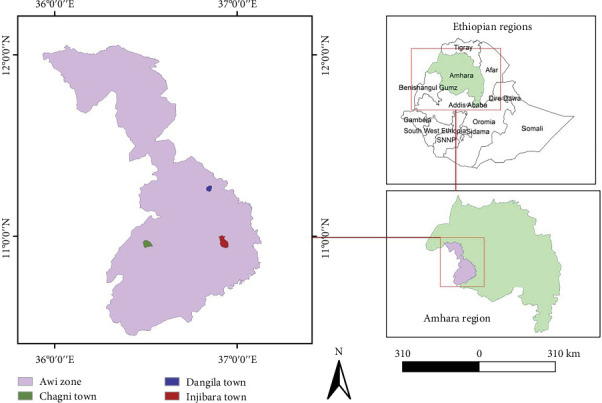
Map of the study area developed by the authors. Source, original data source: Humanitarian Data Exchange (HDX).

The study was conducted in Awi Administrative Zone, Amhara Regional State, Ethiopia (Figure 1). It is located in the southwestern part of the Amhara Region and the northwestern part of Ethiopia. It is about 447 km away from Addis Ababa, the capital city of Ethiopia, and 118 km from Bahir Dar, the capital city of the Amhara Regional State [17]. The administrative center of Awi administrative zone is Injibara, and geographically, Injibara is located at 10°59⁣′N latitude and 36°55⁣′E longitude. The highest and the lowest altitudes of Injibara are 3000 and 2540 m.a.s.l, respectively [18].

According to the 1994 national census, the population of the Awi Administrative Zone was 717,085 in 147,917 households, of whom 357,238 were men and 359,847 were women; and 62,232 or 8.68% of its population were urban dwellers. According to the 2014 Census conducted by the Central Statistical Agency of Ethiopia [19], the population of the Awi Administrative Zone was 982,942, an increase of 37.07% since 1994, of whom 491,865 were men and 491,077 were women. With an area of 9148.43 km^2^, the population density of the Awi Administrative Zone in 2007 was 107.44; and 123,014 or 12.51% were urban inhabitants. A total of 215,564 households were counted in this zone, which results in an average of 4.56 persons per household. The Awi Administrative Zone consists of fast-growing towns, the main ones being Injibara, Dangila, and Chagni. Although the population grew over the years, no change was observed in the waste collection service, which is mainly the municipality's responsibility, to accommodate the increasing population and the complexity of solid waste (degradable and nondegradable). Although the households pay 40 ETB (Ethiopian Birr) ($0.36) for each solid waste collection service, since the municipalities cannot collect the generated solid waste regularly (once a month), the households dispose of the waste in illegal disposal sites like open dump site, rode side, and drainage lines.

### 2.2. Study Design

A community-based cross-sectional study design was used to assess SWM practices and to identify its determinant factors in the Awi Administrative Zone from February 2022 to January 2023. The population that the samples represent consisted of all households in Chagni, Dangila, and Injibara towns in the Awi Administrative Zone.

### 2.3. Sampling Techniques

Three main towns of Awi administrative zone (Chagni, Dangila, and Injibara) were selected to represent the Awi Administrative Zone. Each town has five kebeles (wards, the smallest administrative unit within a town), and one kebele (ward) was randomly selected from each town to represent the town. This is mainly because all five kebeles (wards) in each of the three towns have relatively homogenous characteristics in terms of solid waste type and composition, population density, availability of infrastructures, and lifestyle of the residents. Since the three towns (kebeles (wards)) are internally homogenous (within each town/kebele (wards), the SWM practices are similar) and externally heterogeneous (the towns/kebeles (wards) do not follow the same SWM practices), stratified random sampling method was used (by using the towns/kebeles (wards) as strata) to collect the data from randomly selected households (sampling units).

### 2.4. Sample Size Determination

The total sample size was determined according to Cochran's [20] formula: *n* = *N*/(1 + *N*∗(*e*)^2^) where *n* represents the sample size, *N* is the total number of households across the three kebeles (wards), and *e* denotes the tolerable error, set at 5% (0.05). Data from the administrative offices of each of the selected kebeles (wards) within each town revealed approximately 3354, 2820, and 3255 households in Chagni 01, Dangila 05, and Injibara 01 kebeles (wards), respectively. Thus, *n* = *N*/(1 + *N*∗(*e*)^2^) = 9429/(1 + 9429∗(0.05)^2^) = 340. This sample size (*n* = 340) was then proportionally allocated (*n*_1_ = (*n*∗*n*_1_)/*N*) to the three kebeles (wards) representing the towns, where *n*_*i*_ denotes the sample size of the *i*^th^ kebeles (wards) (*n* = 340), *N*_*i*_ represents the number of households in the *i*^th^ kebeles (wards), and *N* = 9429. Consequently, *n*_1_ = 121 samples were selected from Chagni, *n*_2_ = 102 samples from Dangila, and *n*_3_ = 117 samples from Injibara.

### 2.5. Collection and Sorting of Solid Wastes

The collection and sorting of waste from the participating households took place over 7 consecutive days to ensure an average result for the entire week, accounting for potential differences in waste generation between days. Each household received a plastic bag labeled with its house code number. The following day, during collection, another bag with the same label was provided for the next day's collection, continuing for 7 days. Trained data collectors conducted the collection, each assigned to collect a maximum of solid waste from seven households daily.

### 2.6. Estimating the Generation Rate of Solid Waste

The waste collection from participating households occurred for 7 consecutive days and was weighted using Wholesale Spring Steel Luggage Scales. Subsequently, the average solid waste generated by households each day was calculated by dividing the total solid waste weight within each participant household by 7. The per capita per day solid waste generation rate (PCPDSWGR) is defined as PCPDSWGR = (total solid waste generation within 7 days)/(7 days) × (total family size of survey households) × (total population of the study site) [21].

### 2.7. Data Collection Methods and Survey Instrument

This study utilized a combination of close-ended questionnaires, interviews, and field observations as data collection tools. Following the preparation and translation of the questionnaire into the local language (Amharic), a pilot survey was conducted on randomly selected households outside the sampled kebeles (wards) to address any unclear or misleading questions. Subsequently, the final questionnaire was administered to the sampled households with the assistance of three trained data collectors hired by the researchers. These collectors worked under close supervision to ensure consistency in data collection. Additionally, semistructured and unstructured interviews were conducted with solid waste collection service providers, street sweepers, and staff from the sanitation and beautification department to gather further insights. Furthermore, field observations were done to assess the town's solid waste transportation system, instances of unlawful dumping, home solid waste treatment methods, and types of solid waste collection and segregation. Additionally, pictures were taken during these observations to document disposal sites and instances of illegal dumping.

Household heads were evaluated using 12 practice-related checklists to gauge their SWM practice. Households scoring 9 points or lower (≤ 75%) were categorized as having inadequate (poor) waste management practices, while those scoring above 10 points (> 83%) were classified as exhibiting satisfactory (good) waste management practices [2, 22].

### 2.8. Operational Definitions

Improper SWM refers to inadequate practices in handling solid waste, including failure to utilize proper collection services, improper management or segregation of solid waste, and disposal of generated waste in unauthorized locations.

### 2.9. Data Quality Assurance

The data quality was upheld through meticulous planning and testing of the survey instrument and by equipping the data collectors and supervisors with comprehensive training. The investigator maintained oversight continuously throughout the data collection process to ensure adherence to standard operating procedures. Furthermore, daily checks were conducted to verify the consistency and completeness of the data. To improve clarity, the questionnaires, originally prepared in English, were translated and conducted in Amharic, which is the local language in the study area. Then, the collected data were translated back into English for data entry and analysis.

### 2.10. Data Analysis

The collected data underwent initial manual checks for completeness before being coded and entered into SPSS Version 26.0 for analysis using descriptive statistics and logistic regression modeling. A binary logistic regression model was employed to assess the significance of potential predictors in predicting the outcome variable. Subsequently, predictors deemed significant (with *p* values < 0.05) in the binary logistic regression model were subjected to multivariate analysis to evaluate their individual effects while controlling for other variables. Chi-square (*χ*^2^) tests of independence were conducted to determine the significance of the association between each independent variable and SWM practice (classified as either good or poor). Adjusted odds ratio (AOR) values were reported to indicate the significance of the association between the dependent variable and each factor, elucidating whether the likelihood of achieving a good score increases or decreases when transitioning from the default category (represented as 1.00 in the results) to another category, in the presence of all factors included in the model.

### 2.11. Ethical Considerations

Ethics approval and consent to participate are not required for this study, as it did not involve the use of names of individual participants. However, the researcher obtained permission to conduct the study through a written letter from Injibara University (a newly established university in Ethiopia). This permission letter, written in Amharic, authorized the research to be carried out.

Furthermore, participants were briefed on the study's objectives, and their verbal consent was sought. Their right to decline participation or withdraw from the study at any point was fully respected, and the confidentiality of the information shared by each participant was rigorously upheld.

## 3. Results

### 3.1. Descriptive Statistics of Sociodemographic Characteristics

A total of *n* = 340 households participated (100% response rate) in the study. Of the total respondents, 228 (67.1%) were female and 112 (32.9%) were male. Fifty-nine (17.4%) of the respondents had completed their first degree or above, 84 (24.7%) of them had a certified diploma, and the remaining 57.9% completed high school or below. Regarding their age group, the majority of the respondents, 178 (52.4%), were between 31 and 50 years old. Regarding family size, 16 (4.7%) had either one- or two-family size, 133 (39.1%) respondents had a family size of three to four, 129 (37.9%) of them had a family size of five to six, and the remaining 18.2% had a family size of seven or more. The monthly income of 197 (57.9%) respondents was between 2000 and 5000 ETB, which is equivalent to between $31 and $78. Only 40.9% of the participant households followed good SWM practices (Table 1).

### 3.2. Institutional (Municipality) Solid Waste Collection Service

Regarding the accessibility of solid waste collection service, 138 (40.6%) households had access to the service in a fragmented way; and 35.9% and 26.2% of the households disposed their solid waste in riverside bridge/drainage line and roadside/open land (Appendix S1, Figures S1 and S4), respectively. These are illegal disposal sites. Only 39 (11.5%) of the households received information about where to dispose of the waste and about the punishment if they dispose of their generated solid waste at an illegal disposal site, but the other (88.5%) households were not aware of illegal SWM rules and regulations in the Awi Administrative Zone.

During key informants' interview, the head of the town's sanitation and beautification department said that “lack of community awareness and budget constraints (i.e., the budget assigned to solid waste management department) made it hard to achieve this critical service properly.” According to their statement, “to address budget limitations in the study area, the head of the town's Sanitation and Beautification department is attempting to collect 40 ETB (approximately $0.40) from households receiving municipal tap water to support solid waste management efforts. The lack of human resources in the department is another reason that made it difficult to create awareness and/or control illegal disposal of solid waste management practices. Also, the little or no attention given by the local political leaders to solid waste management service was a challenge that hinders effective delivery of this service in Awi administrative zone.”

Our field observations revealed that the solid waste collection and transport system is not well organized and equipped with solid waste collection materials and technologies. The solid waste collectors suffer from a lack of financial and moral support from the community and government agencies. Also, since the collectors do not have vehicles to collect and transport the waste, they use pushcarts and horse carts (Appendix S1, Figure S2). As a result, private solid waste collectors were not engaged in collecting solid waste according to the regular collection interval. Additionally, the study area lacks a designated, legally isolated disposal site, with the temporary waste disposal facility being located close to residential neighborhoods, public spaces, and social service organizations.

The study also revealed that 41.8% of the households did not know the presence of cleanup campaigns in their town, and they did not participate in any campaign. The remaining 58.2% of the households participated in the cleanup campaign programs in the Awi Administrative Zone.

### 3.3. Determination of Solid Waste Generation Rate

The per capita daily solid waste generation rates at the household level for the selected kebele (wards) in Injibara, Dangila, and Chagni were 0.443, 0.456, and 0.487 kg/capita/day, respectively. Using these rates, the daily total solid waste generation for the residential areas of the selected kebele (wards) was calculated by multiplying the total population of Injibara 01 kebele (wards), Dangila 05 kebele (wards), and Chagni 01 kebele (wards) (48,163, 44,949, and 50,629) by the per capita household solid waste generation rate. Consequently, the daily total solid waste generation for Injibara 01 kebele (wards), Dangila 05 kebele (wards), and Chagni 01 kebele was determined to be 21.34, 20.50, and 24.67 tons, respectively (Table 2).

### 3.4. Factors Associated With SWM Practices in Awi Administrative Zone

The test of independence results shown in Table 3 indicate that the education level, number of family members, access to solid waste collection service, reduction of solid waste from the source, and awareness about the environmental and human health impacts of improper SWM are significantly associated with waste management practice.

The binary logistic regression results reported in terms of the AOR (Table 3) show that the age of the household head, family size, monthly income level, solid waste selling practice, reuse of solid waste, following rules and regulations during disposal of solid waste, frequency of participation in cleanup campaigns, and awareness about the environmental and human health impacts of improper SWM were significant factors that affect the level of SWM practice in the Awi Administrative Zone.

Compared to older (above 50 years old) heads of households, 31–50-year-old heads of households are 66% less likely to follow proper SWM practice (AOR = 0.34). Compared to households whose family size is 7 or more, those who had 3–4 and 5–6 family sizes are 8.5 and 4.3 times more likely to follow proper SWM practice in the Awi Administrative Zone, respectively (Table 2). Household heads who have low (< 2000 ETB) monthly income are 5.3 times more likely to follow good SWM practice than household heads who have high (> 5000 ETB) monthly income. The households who sell solid waste are 54% less likely to follow improper SWM practice (AOR = 0.46) than those who do not. Households who practice reusing solid waste at the source are 74% less likely to follow improper SWM practice than those who do not reuse solid waste (AOR = 0.26). Households who have awareness about applying rules and regulations during the illegal disposal of solid waste were 94% less likely to practice improper SWM practice (AOR = 0.06) than those who are not sure whether they are applying rules and regulations during illegal disposal of solid waste. Households who participated once in the cleanup campaign during the previous year were 77% less likely to practice proper SWM when compared to those who participated two or more times (AOR = 0.23). Households who had awareness and were slightly aware of the environmental and human health impacts of improper SWM were 9.5 times and 3.6 times, respectively, more likely to have good SWM practices when compared to those households who do not have awareness (Table 3).

## 4. Discussion

SWM has become a critical concern for many developing countries including Ethiopia. In Ethiopia, SWM is mainly the responsibility of municipalities, but this service was not adequately delivered in most parts of the country [23]. In many towns in Ethiopia, waste management practices are poor and only around 2% of the population get solid waste collection, transportation, and disposal services [8].

The estimated average daily solid waste generation rates per person in Injibara, Dangila, and Chagni towns were 0.443, 0.456, and 0.487 kg/capita/day, respectively. This finding aligns with the findings of previous studies on solid waste generation rates, which reported rates of 0.4 kg/capita/day [24], 0.43 kg/capita/day in Gondar town [9], 0.45 kg/capita/day in Dessie City [4], and 0.542 kg/capita/day [25] in Addis Ababa, Ethiopia. Similarly, studies conducted by the Dire Dawa City Health Office indicated a solid waste generation rate of 0.5 kg/capita/day. The similarity in estimated solid waste generation rates may be attributed to different factors such as population density, consumption patterns, lifestyle behaviors, and economic status of the participants.

The current solid waste generation rate in the study area closely mirrors that of previous studies conducted in cities across Ethiopia, indicating a trend of increasing waste generation rates in urban areas. This increase is attributed to population growth, rapid urbanization, and improved living standard factors. Solid waste generation in most developing country cities was estimated to be between 0.4 and 0.6 kg/capita/day [4], which closely aligns with the current study's findings on the solid waste generation rates of Injibara, Dangila, and Chagni towns.

Different types of solid waste were reported to have been generated by households. Based on field observation and segregation of the generated solid waste, the majority of the generated solid wastes were microbial degradable solid waste (Appendix S1, Figure S3) like cooking, agricultural, and sweeping solid wastes, which is similar to what was reported in Assela [13] and Dire Dawa towns [26], Ethiopia. This might be due to the similar socioeconomic status, educational background, lifestyle, and availability of local materials. In addition, plastic shopping bags locally known as “festal” [27] waste were also generated in all three selected towns of the Awi Administrative Zone. Moreover, in Chagni town, most households generate plastic bottles (Appendix S1, Figure S4). This is mainly because the city is relatively dry, and the community needs lots of water and fluids to drink. After using sealed or canned drinks, people dispose the plastic bottles in open dump sites because of lack of waste management facilities.

Many households collect their home garbage in plastic bags and dispose of it in open fields. Some people collect used plastic bottles and metallic wastes informally and sell them to recycling businesses to get money in the Awi Administrative Zone. Although informal plastic collectors provide a substantial positive contribution to the country's management of plastic trash, they are not given enough attention [27]. Additionally, the Awi Administrative Zone is abundant in bamboo cultivation, with some residents opting to use locally crafted bamboo shopping bags as an alternative to plastic ones. To reduce the high usage of plastic bags, promoting and supporting the production and use of biodegradable bags made from bamboo trees is a good idea.

In this study, the lack of solid waste collection service caused households to dispose of the generated solid waste at improper disposal sites like riversides, bridges, and drainages, as well as in roadside and open lands (62.1%). This number is similar to that reported for Ethiopia and Tanzania where 75% and 62% of households, respectively, disposed of their solid waste in unauthorized sites [28, 29]. This might be due to the similarity of poor solid waste collection service provided by the town municipalities, availability of infrastructure, lack of community awareness on the impacts of improper SWM on the environment and human health, and lack of enforcement of SWM regulations in Ethiopia and in Tanzania. In addition to the poor solid waste collection service, only 11.5% of the households were aware of the rules and regulations of SWM. The awareness of SWM in this study was lower than that reported for Gondar [30] and for Bahir Dar [31], Ethiopia. This difference in awareness might be due to the involvement of different nongovernmental organizations like Dream Light Private Limited Company in Bahir Dar, which is highly engaged in proper SWM awareness creation and waste collection service among the community [31].

Among the respondents of this study, 35.9% of them dumped their solid waste improperly on riverside bridge/drainage lines, and 26.2% dumped on roadside/open land; this disposing practice shows that most of them are not aware of proper SWM, which calls for awareness creation. In public health, the consequences of these statistics could include increased risk of environmental pollution, water contamination, and the spread of diseases. Improper solid waste disposal in riverside bridges/drainage lines and roadside/open land can lead to the contamination of water sources, breeding of disease-carrying vectors, and overall degradation of the environment. Lack of proper waste management practices can contribute to public health hazards and impact the well-being of individuals living in these areas. Efforts to improve waste collection services including door-to-door collection service and promote responsible waste disposal practices are essential to mitigate these negative consequences on public health.

In this study, gender, access to community bins, who usually takes the solid waste, ways/sites of disposing of solid waste, cleanup campaign frequency, and participation in cleanup campaigns had no significant relationship with SWM practice. These findings are consistent with those reported for Assela town, Ethiopia [13]. This consistency might be explained by the similarity of the door-to-door solid waste collection service, town infrastructure, and SWM willingness of the households.

According to the logistic regression results, 31–50-year-old respondents are 66% less likely to follow proper SWM practice (AOR = 0.34). According to the response of key informants, this is because many of the older households live in their own house and they try to keep their village beautiful to attract new residents and house renters than younger respondents. Households who had 3–4 and 5–6 family sizes are 8.5 and 4.3 times more likely to follow proper SWM practices than households with 7 or more family sizes. This is due to the amount of generated solid waste, since households with 3–4 family size generate smaller amount of solid waste that can be managed easily than those who have more than 7 family size. Similarly, in this study, households who have low (< 2000 ETB) monthly income level are 5.3 times more likely to follow good SWM practices than household heads who have high (> 5000 ETB) monthly income. This is because households with high monthly income levels may generate more solid waste, and with a lack of awareness, they may dispose of the waste improperly in an illegal dump site. Respondents who practice reusing solid waste at the source are 74% less likely to follow improper SWM practices than those who do not reuse solid waste (AOR = 0.26). This may be due to the lower amount of generated solid waste and the conversion of waste to income, which leads to the reduction of disposed solid waste in illegal dumping sites.

Our study showed that 59.1% of households practice improper SWM in the Awi Administrative Zone. However, this finding is not consistent with the results of studies conducted in other parts of Ethiopia (Assela 82.8% [13] and Fiche 78.4% [2]), in Nigeria (83.3%) [32], and in Ghana (81.7%) [33]. Possible explanations for this difference might be differences in the lifestyle of the households, sociodemographic characteristics of households, and infrastructure differences that affect the percentage of proper SWM practices. However, the level of improper SWM practice in the Awi Administrative Zone is similar to that in other towns of Ethiopia (Gondar: 69.7% [30], Dire Dawa: 68.4% [26], and Debre Berhan 67.4% [34]) and in Uganda (58.7%) [22]. This might be due to the similarity of sociodemographic, lifestyle, solid waste collection service, and governmental interventions to improve SWM and enforcement of waste management rules and regulations.

This study also showed a relatively low access to solid waste collection service (40.6%) compared to that in Addis Ababa (84%) [24]. This difference in the accessibility of solid waste collection services might be due to a lack of infrastructure in the Awi Administrative Zone compared to that in Addis Ababa, which calls for the municipalities to increase their budget for infrastructure. Also, the solid waste collection services in Addis Ababa are accessible to most of the households who are willing to pay [24, 35]. Similar studies conducted in Mombasa, Kenya [13], and Adama, Ethiopia [36], showed the presence of better solid waste collection services.

The present study demonstrated the lack of adequate door-to-door solid waste collection service and sufficient community awareness regarding the regulations and guidelines on SWM. It also revealed that reduce, reuse, and recycle (the three Rs) practices are deficient in the study area. Consequently, the residents are engaged in inappropriate disposal of the generated solid waste in unauthorized locations. These activities negatively impact both the environment and human health. To address this predicament effectively, it is imperative to enhance awareness among the local community by offering appropriate training.

### 4.1. Limitations

First, the collected data are from a sample of households and not from the entire population. Although a random sampling method was used to avoid bias, there is always unavoidable sampling error. Second, generalizability is also a limitation because this study focused on the SWM practices of only three towns of the Awi Administrative Zone. Third, the study solely focused on SWM and did not consider the management practices of liquid waste within the study area.

## 5. Conclusion

This study revealed the presence of poor SWM practices in the Awi Administrative Zone. Poor SWM is practiced mainly by younger heads of households, households with larger family size, households with higher monthly income, households who do not sell solid waste, households who do not reuse solid waste, households who do not adhere to the rules and regulations of solid waste disposal, households who do not participate in cleanup campaigns, and households who do not understand the environmental and human health impacts of improper SWM.

This study contributes to the understanding of waste management by identifying key influencing factors and challenges, offering policy recommendations, emphasizing the link between SWM and public health, promoting community engagement, providing a framework for future research, and proposing practical solutions for improving waste management practices in the region. Based on the findings of this study, we make the following practical recommendations to improve the town's SWM system: (1) The municipalities need to be engaged in effective and regular door-to-door solid waste collection service. (2) There is a need for awareness creation to the local community by governmental and nongovernmental institutions to encourage proper SWM practices. (3) The municipalities need to pay close attention on how to overcome budget constraints for providing facilities (like modern means of solid waste transportation) and to support unemployed women and young people for starting SWM enterprises that can work with the municipalities to provide door-to-door solid waste collection services regularly. (4) To address issues related to intentional and illegal garbage dumping in the community, the municipalities need to enforce the rules and regulations and (5) promote the use of locally crafted bamboo shopping bags as a sustainable alternative to plastic bags.

## Figures and Tables

**Table 1:** Sociodemographic characteristics and household frequencies in Awi Administrative Zone, northwestern Ethiopia (*n* = 340).

**Variable/category**	**Freq.**	**%**
*Household gender*		
Male	112	32.9
Female	228	67.1
*Household head's age (years)*		
18–30	84	24.7
31–50	178	52.4
Above 50	78	22.9
*Household head's education*		
Cannot read and write	36	10.6
Can read and write but have no formal education	48	14.1
Completed Grades 1–4	29	8.5
Completed Grades 5–8	39	11.5
Completed Grades 9–12	45	13.2
Received diploma	84	24.7
Received first degree or above	59	17.4
*Family size*		
1–2	16	4.7
3–4	133	39.1
5–6	129	37.9
7 or more	62	18.2
*Monthly income (ETB)*		
< 2000	74	21.8
2000–5000	197	57.9
> 5000	69	20.3
*Access to shared containers (community bins)*		
Yes	67	19.7
No	273	80.3
*Who collects the solid waste?*		
A member of the household	148	43.5
Microenterprise	90	26.5
Daily laborer	102	30.0
*Ways/sites of disposing of solid waste*		
Throwing to riversides/bridges/drainages	122	35.9
Throwing to roadsides/open land	89	26.2
Burning	79	23.2
Waiting until waste collectors come back	50	14.7
*Sell recyclable solid waste*		
Yes	146	42.9
No	194	57.1
*Access to solid waste collection service*		
Yes	138	40.6
No	202	59.4
*Reuse solid waste*		
Yes	169	49.7
No	171	50.3
*Followed rules and regulations when disposing of solid waste*		
Yes	39	11.5
No	127	37.4
I do not know	174	51.2
*Frequency of cleanup campaigns at the village level*		
Rare	127	37.4
Weekly	8	2.4
Monthly	63	18.5
I do not know	142	41.8
*Participated in cleanup campaigns*		
Yes	198	58.2
No	142	41.8
*Cleanup campaign participation per year*		
None	142	41.8
One time	104	30.6
Two or more times	94	27.6
*Awareness about the impacts of improper solid waste management on the environment and health*		
Aware	123	36.2
Slightly aware	86	25.3
Unaware	131	38.5
*Level of solid waste management practice*		
Good	139	40.9
Poor	201	59.1

**Table 2:** Household solid waste generation rate in Awi Administrative Zone, northwestern Ethiopia.

**District**	**kg/capita/day**	**Population size**	**Tons/day**	**Tons/week**	**Tons/month**	**Tons/year**
Injibara (01 kebele)	0.443	48,163	21.34	149.35	640.09	7681.04
Dangila (05 kebele)	0.456	44,949	20.50	143.48	573.91	6886.91
Chagni (01 kebele)	0.487	50,629	24.66	172.59	690.38	8284.52

**Table 3:** Chi-square (*χ*^2^) test of independence between categorical variables and solid waste management practices in Awi Administrative Zone, northwestern Ethiopia.

**Variable/category**	**Solid waste management practice**		**χ** ^2^ ** test ** **p** ** value**	**AOR**	**p** ** value**
	**Good (** **n** = 139**)**	**Poor (** **n** = 201**)**			
*Household gender*			0.091		
Male	53	59		0.878	0.686
Female	86	142		1.00	
*Household age (years)*			0.306		
18–30	39	45		0.421	0.111
31–50	66	112		0.335	0.014⁣^∗^
> 50	34	44		1.00	
*Household education level*			< 0.001		
Cannot read and write	16	20		2.350	0.312
Can read and write but no formal education	12	36		0.986	0.987
Completed Grades 1–4	9	20		2.019	0.419
Completed Grades 5–8	9	30		1.545	0.617
Completed Grades 9–12	15	30		1.085	0.920
Received diploma	48	36		1.381	3.624
Received first degree or above	30	29		1.00	
*Family size*			0.002		
1–2	8	8		3.429	0.138
3–4	69	64		8.520	< 0.001⁣^∗^
5–6	46	83		4.264	0.004⁣^∗^
7 or more	16	46		1.00	
*Monthly income (ETB)*			0.117		
< 2000	38	36		5.252	0.004⁣^∗^
2000–5000	74	123		3.912	0.107
> 5000	27	42		1.00	
*Access for shared community bins*			0.344		
Yes	24	43		1.593	0.278
No	115	158		1.00	
*Who collects the solid waste*			0.573		
A member of the household	61	87		1.092	0.784
Microenterprise	33	57		0.679	0.304
Daily laborer	45	57		1.00	
*Ways of disposing solid waste*			0.498		
Throwing to riversides/bridges/drainages	45	77		1.560	0.322
Throwing to roadsides and open land	42	47		1.385	0.477
Burning	31	48		1.187	0.725
Waiting until waste collectors come back	21	29		1.00	
*Sell solid waste*			0.878		
Yes	59	87		0.460	0.015⁣^∗^
No	114	80		1.00	
*Access to solid waste collection service*			0.005		
Yes	44	94		1.476	0.408
No	95	107		1.00	
*Reused solid waste*			< 0.001		
Yes	47	122		0.256	0.008⁣^∗^
No	92	79		1.00	
*Follow solid waste disposal rules and regulations*			0.516		
Yes	13	26		0.064	< 0.001⁣^∗^
No	51	76		0.551	0.134
I do not know	75	99		1.00	
*Cleanup campaign frequency*			0.097		
Rare	51	76		0.352	0.272
Weekly	2	6		0.387	0.486
Monthly	19	44		0.339	0.268
I do not know	67	75		1.00	
*Participated in cleanup campaigns*			0.269		
Yes	76	122		0.566	0.685
No	63	79		1.00	
*Participation time (frequency per year)*			0.377		
No one	63	79		0.141	0.110
One time	37	67		0.227	0.009⁣^∗^
Two and more times	39	55		1.00	
*Awareness about the environmental and health impacts of improper solid waste management*			< 0.001		
Aware	74	49		9.502	< 0.001⁣^∗^
Slightly aware	33	53		3.554	0.030⁣^∗^
Unaware	32	99		1.00	

## Data Availability

The data that support the findings of this study are available from the corresponding author upon reasonable request.

## Supporting Information

Supporting Information Additional supporting information can be found online in the Supporting Information section. The initial collection of data and information was done through the administration of a questionnaire. Furthermore, onsite observations were carried out, in conjunction with accompanying images as references. The relevant figures have been included in the supporting information of the system. Appendix S1. Figure S1: Distribution of illegal disposal sites in the Awi Administrative Zone (photo by Belsti Atnkut). Description: This figure illustrates the geographical distribution of illegal disposal sites within the Awi Administrative Zone, highlighting areas with high concentrations of unauthorized waste disposal. Figure S2: Proportion of horse cart transportation mechanism for solid waste in the Awi Administrative Zone (photo by Belsti Atnkut). Description: This figure shows the percentage breakdown of solid waste transportation using horse carts as a mechanism in the Awi Administrative Zone, indicating the extent of reliance on this traditional method. Figure S3: Composition of microbial degradable solid waste generated in the Awi Administrative Zone (photo by Belsti Atnkut). Description: This figure illustrates the breakdown of microbial degradable solid waste generated within the Awi Administrative Zone. This composition provides a detailed overview of the types of waste that undergo microbial degradation processes in the region, offering valuable insights into waste management strategies and environmental considerations within the area. Figure S4: Composition of microbial nondegradable solid waste generated in the Awi Administrative Zone (photo by Belsti Atnkut). Description: The figure shows the composition of microbial nondegradable solid waste generated in the Awi Administrative Zone. This breakdown offers a comprehensive understanding of the types of waste that do not readily undergo microbial degradation processes within the region. By highlighting these nondegradable components, the figure sheds light on potential challenges in waste management and environmental sustainability efforts within the area.
