# Supplementary material for: Factors Influencing Solid Waste Management Practices and Challenges in Awi Administrative Zone, Northwestern Ethiopia
Source: Biomed Res Int. 2025 Jan 29;2025:1311674. doi: 10.1155/bmri/1311674 (PMC11824480; doi:10.1155/bmri/1311674)
Supplement: Supporting Information — Additional supporting information can be found online in the Supporting Information section. The initial collection of data and information was done through the administration of a questionnaire. Furthermore, onsite observations were carried out, in conjunction with accompanying images as references. The relevant figures have been included in the supporting information of the system. Appendix S1. Figure S1: Distribution of illegal disposal sites in the Awi Administrative Zone (photo by Belsti Atnkut). Description: This figure illustrates the geographical distribution of illegal disposal sites within the Awi Administrative Zone, highlighting areas with high concentrations of unauthorized waste disposal. Figure S2: Proportion of horse cart transportation mechanism for solid waste in the Awi Administrative Zone (photo by Belsti Atnkut). Description: This figure shows the percentage breakdown of solid waste transportation using horse carts as a mechanism in the Awi Administrative Zone, indicating the extent of reliance on this traditional method. Figure S3: Composition of microbial degradable solid waste generated in the Awi Administrative Zone (photo by Belsti Atnkut). Description: This figure illustrates the breakdown of microbial degradable solid waste generated within the Awi Administrative Zone. This composition provides a detailed overview of the types of waste that undergo microbial degradation processes in the region, offering valuable insights into waste management strategies and environmental considerations within the area. Figure S4: Composition of microbial nondegradable solid waste generated in the Awi Administrative Zone (photo by Belsti Atnkut). Description: The figure shows the composition of microbial nondegradable solid waste generated in the Awi Administrative Zone. This breakdown offers a comprehensive understanding of the types of waste that do not readily undergo microbial degradation processes within the region. By highlighting these nondegradable [file 1311674.f1.docx]

**Appendix 1:** Distribution of illegal disposal sites, proportion of horse cart transportation mechanism for Solid Waste and composition of solid waste generated in Awi Administrative Zone.


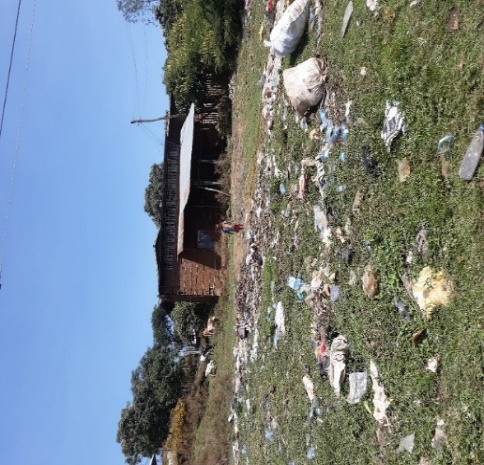

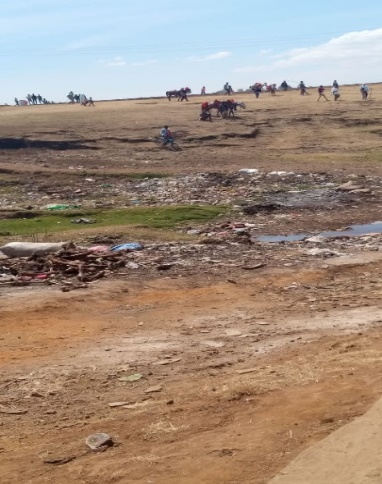

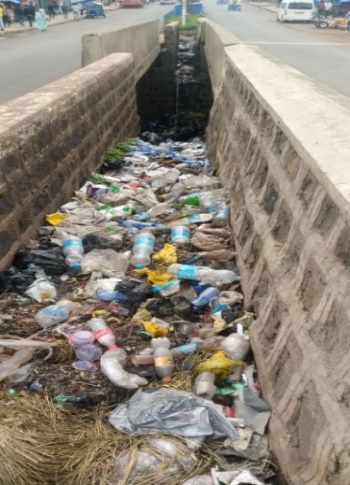

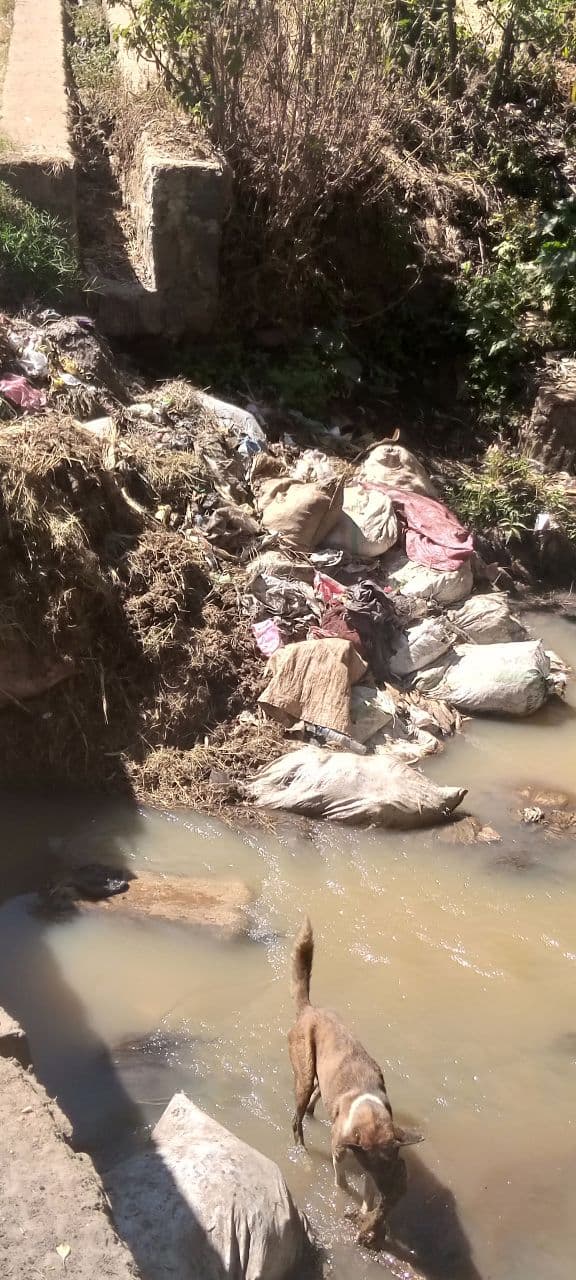

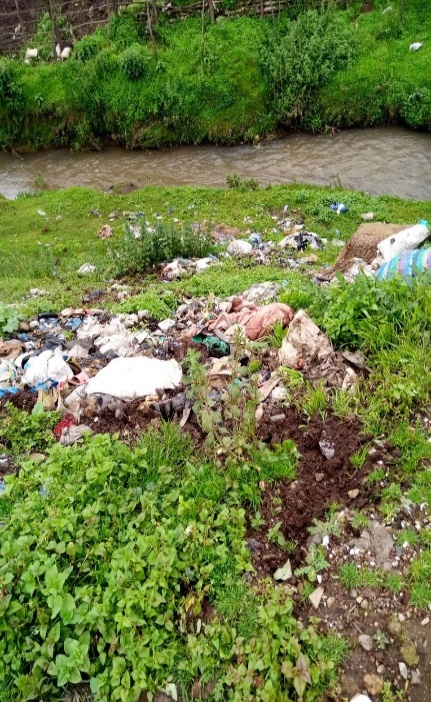


**Figure 1.** Distribution of illegal disposal sites in Awi Administrative Zone (Photo by Belsti Atnkut).

**Description:** This figure illustrates the geographical distribution of illegal disposal sites within the Awi administrative zone, highlighting areas with high concentrations of unauthorized waste disposal.


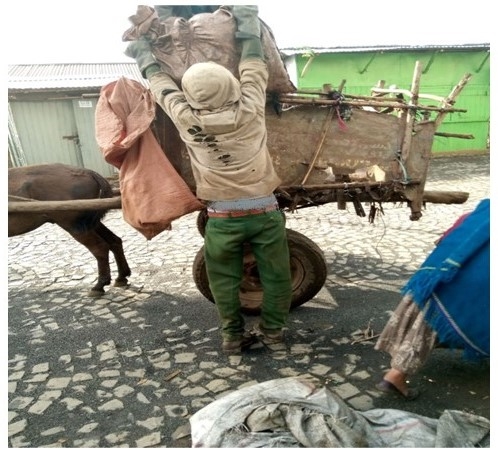


**Figure 2:** Proportion of horse cart transportation mechanism for solid waste in Awi Administrative Zone (Photo by Belsti Atnkut).

**Description**: This figure presents the percentage breakdown of solid waste transportation using horse carts as a mechanism in the Awi administrative zone, indicating the extent of reliance on this traditional method.


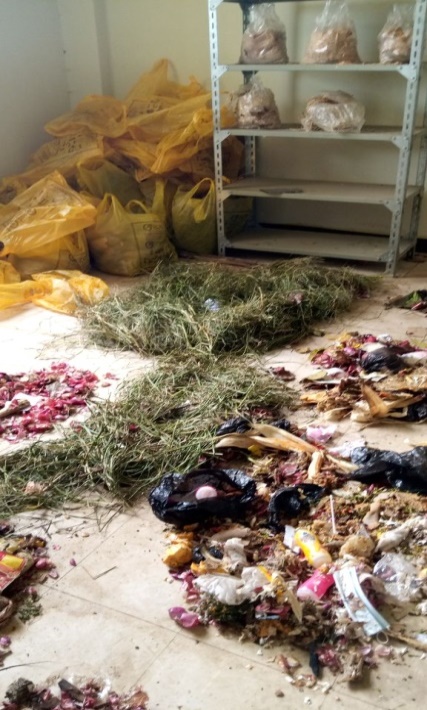

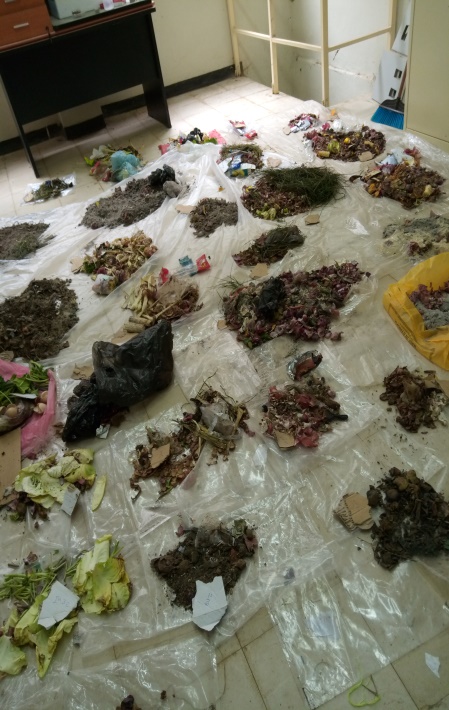

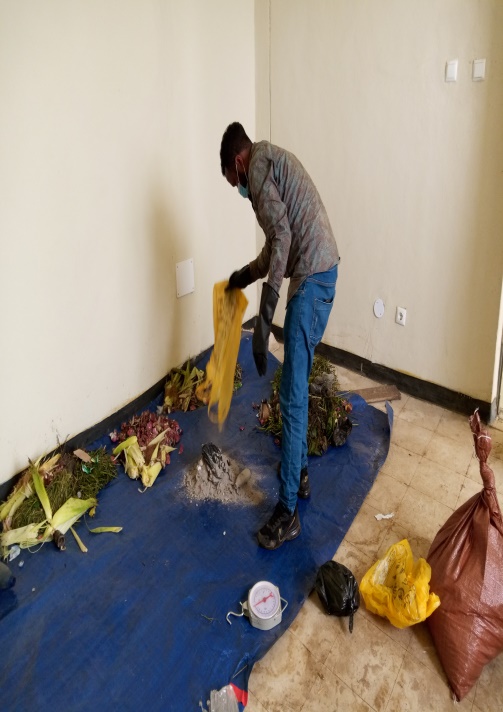


**Figure 3:** Composition of microbial degradable solid waste generated in Awi Administrative Zone (Photo by Belsti Atnkut).

**Description**: This figure illustrates the breakdown of microbial degradable solid waste generated within the Awi Administrative Zone. This composition provides a detailed overview of the types of waste that undergo microbial degradation processes in the region, offering valuable insights into waste management strategies and environmental considerations within the area.

**Figure 4.** Composition of microbial non-degradable solid waste generated in Awi Administrative Zone (Photo by Belsti Atnkut).


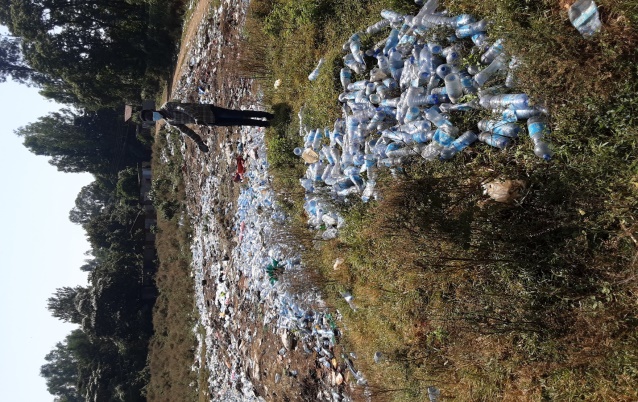

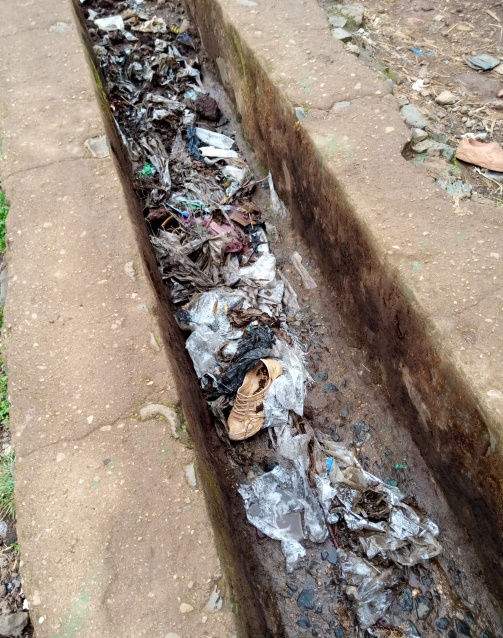

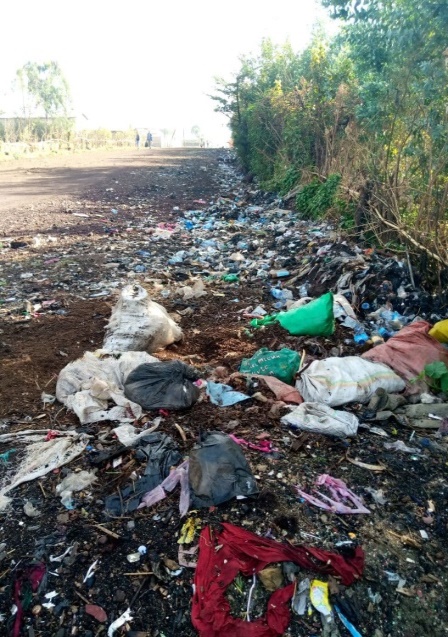


**Describe**: The figure represents the composition of microbial non-degradable solid waste generated in the Awi Administrative Zone. This breakdown offers a comprehensive understanding of the types of waste that do not readily undergo microbial degradation processes within the region. By highlighting these non-degradable components, the figure sheds light on potential challenges in waste management and environmental sustainability efforts within the area.
